# Robust single-mode laser via merging bound state in the continuum

**DOI:** 10.1038/s41377-026-02355-w

**Published:** 2026-05-27

**Authors:** Kai Peng, Jiyoung Moon, Yilin Meng, Kiyanoush Goudarzi, Wei Li, Qing Gu, Wei Bao

**Affiliations:** 1 Department of Materials Science and Engineering, Rensselaer Polytechnic Institute, Troy, NY USA; 2 Department of Electrical and Computer Engineering, North Carolina State University, Raleigh, 27695 NC USA; 3 Department of Physics, North Carolina State University, Raleigh, 27695 NC USA

**Keywords:** Lasers, LEDs and light sources, Photonic crystals

## Abstract

Bound states in the continuum (BICs) are optical states that remain perfectly confined despite existing within the radiation spectrum, enabling strong light confinement and light-matter interactions. These unique properties make BICs a promising platform for high-performance photonic crystal lasers. However, achieving robust, single-mode BIC lasers with compact footprints remains challenging due to mode competition and fabrication imperfections. Here, we demonstrate a robust single-mode laser by leveraging the concept of “merging BIC” in momentum space, which enables stable lasing behavior up to 80 times the threshold power, showcasing exceptional mode stability under high-power excitation. Furthermore, we realize an ultra-compact photonic crystal laser by combining the BIC mode with edge engineering, achieving a 5 × 5 periodic array in which the entire patterned photonic crystal region has an area smaller than 15 μm². These results provide a promising pathway toward high-performance, miniaturized lasers for photonic applications.

## Abstract (web summary)

BIC-merging physics enables robust single-mode lasing in ultra-compact photonic crystal laser devices.

## Graphical abstract

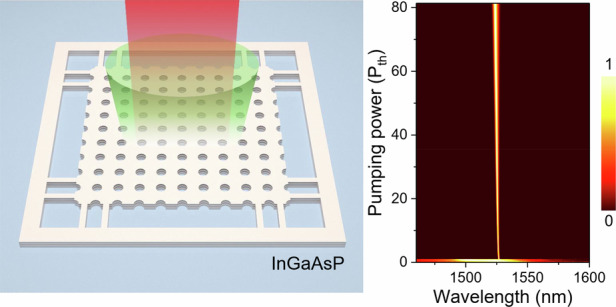

## Introduction

Robust single-mode operation is one of the most desirable features of compact semiconductor lasers, especially for integrated photonic applications requiring spectral purity, energy efficiency, and stability under high-power operation. However, achieving such performance in a miniaturized footprint remains a persistent challenge due to intrinsic trade-offs among cavity size, quality factor (Q), and mode selectivity. Among various candidates, photonic crystal surface-emitting lasers offer numerous advantages, including high-power output, near-diffraction-limited low beam quality factor (M^2^) output, small divergence angles, low spectral dependence on temperature, compact device structures, and the ability to support ultrafast beam steering of the emitted light^1^. However, mode competition in photonic crystal lasers leads to multimode emission under high-power operation. To overcome this challenge, various mechanisms have been proposed for mode selection^2–10^, with the ultimate goal of realizing stable single-mode operation with high output power.

Bound states in the continuum (BICs), originally proposed in quantum mechanics^11^, have recently emerged as a powerful tool for creating high-performance nanophotonic devices^12–14^. By suppressing radiative losses through interference between different resonant modes, BICs are predicted to possess an infinite Q-factor, and are closely connected to momentum-space polarization vortices carrying topological charges^15–18^. Building on these properties, BICs have enabled a broad range of nanophotonic functionalities, including high-Q resonances^19–28^, chiral emission^29–32^, and enhanced light-matter interactions^33–35^. Notably, BIC concepts have also been exploited to realize photonic-crystal lasers with reduced radiative loss and low thresholds^35–43^. However, the performance of BIC modes can drastically decline when finite-size perturbations disrupt the optical cavity. High-Q modes are also susceptible to fabrication imperfections and often coexist with competing modes, making stable single-mode lasing difficult to achieve. Furthermore, the growing need for integration and scalability drives the demand for BIC lasers with even smaller footprints. Although concepts such as super-BICs^39^ and mini-BIC cavities^38,41^ have been proposed and demonstrated to achieve notable threshold reduction and footprint minimization, a strategy for sustaining robust single-mode operation over a wide pump range in finite BIC lattices is still lacking.

In this work, we demonstrate a robust single-mode surface-emitting laser enabled by the merging of individual BICs in momentum space. Photonic crystal slabs are known to support not only symmetry-protected BICs at the Γ point, but also accidental BICs with different wave vectors within the same photonic band^19,44^. By tailoring the structure, multiple accidental BICs can be shifted in momentum space and merged with the symmetry-protected BIC at the Γ point^27,45^. The resulting merging BIC exhibits a significantly higher Q-factor and reduced radiative losses than an isolated BIC. Leveraging this effect, we realize a merging BIC laser that operates with a substantially lowered lasing threshold. Notably, at the pre-merging condition, single-mode operation persists up to 80 times the lasing threshold, significantly enhancing stability under high excitation, a general effect previously never discovered. Furthermore, we achieve an ultra-compact photonic crystal BIC laser featuring a device of only 5 × 5 periods (~15 μm²), demonstrating the feasibility of ultra-compact BIC-based on-chip lasers. Band structure measurements and far-field interference patterns confirm the BIC nature of the lasing mode. Our results establish a promising strategy for realizing highly stable, miniaturized single-mode lasers for integrated photonic applications.

## Results

### Theoretical analysis of the merging BIC laser

**Fig. 1:**
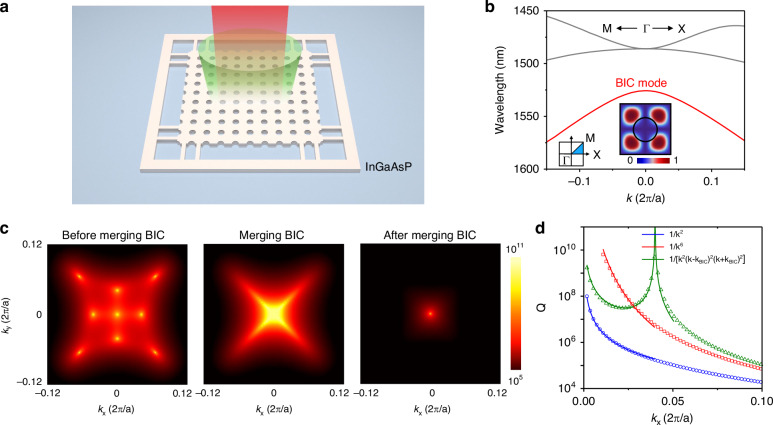
Theoretical analysis of the merging bound state in the continuum (BIC) laser. **a** Schematic representation of the photonic crystal slab and the emission from the BIC mode. **b** Simulated band structure of the photonic crystal devices. The BIC band is indicated by a red line, and the inset shows the top-view electric field distribution (at the center plane of the quantum well) of the BIC mode at Γ point in a unit cell. The first Brillouin zone of the square lattice is also shown in the inset. **c** Simulated far-field Q-factor distribution of the BIC modes, illustrating the evolution from before merging, to at the merging point, and after merging cases. As the hole diameters of the photonic crystal slab increase, the eight isolated off-Γ BICs move toward the at-Γ BIC and merge into a single BIC. Simulation parameters: 940 nm period, and air hole diameters are 484, 488, and 510 nm for before merging, merging, and after merging BIC scenarios, respectively. **d** Q-factor scaling along the ΓX direction of the BIC mode before merging (green), at the merging point (red), and after merging (blue). The merging BIC case (red line and dots), characterized by a high Q-factor near the Γ point, follows a scaling behavior of $Q\propto 1/{k}^{6}$, whereas the before and after merging cases follow $Q\propto 1/{k}^{2}$

Figure 1a shows the schematic of our merging BIC laser. We designed a two-dimensional square lattice air-hole photonic crystal using InGaAsP multiple quantum well gain with a thickness of 320 nm, operating near the telecommunication wavelength (λ ~ 1.55 μm). Three-dimensional finite element simulations (commercial COMSOL Multiphysics software package) of the infinite-sized cavity show three high-Q modes near the designed wavelength, residing in three bands along ΓX and ΓM directions, depicted in Fig. 1b. BIC modes reside in the band marked by the red solid line. The inset shows the electric field distributions of the BIC mode at the Γ point within a single unit cell.

Figure 1c shows the Q-factor distribution of the band in k-space. Generally, there exists a symmetry-protected BIC at the Γ point along with eight accidental BICs. These nine BIC modes exhibit topological charges of ±1, as illustrated in Fig. S2. As the size of the air holes of the photonic crystal slab increases, the eight off-Γ accidental BICs gradually move towards the Γ point in momentum space and finally merge with the at-Γ symmetry-protected BIC. Upon further increasing the diameter of the air holes past the merging BIC condition, a single BIC at the Γ point remains. This merging of BICs significantly impacts the overall loss at the BIC resonance^27^. In the case of an isolated BIC mode, the Q-factor exhibits a quadratic decay in k-space; while in the merging scenario, this scaling transitions to 1/*k*^6^, as depicted in Fig. 1d. This behavior creates a region of higher Q-factor around the Γ point for the merging BIC, with the Q-factor decreasing more gradually away from the Γ point compared to that of an isolated BIC mode. Such characteristics suggest an increased robustness against symmetry-breaking perturbations and improved tolerance to nanofabrication variations.

### BIC laser performance of the 20 × 20-period devices

**Fig. 2:**
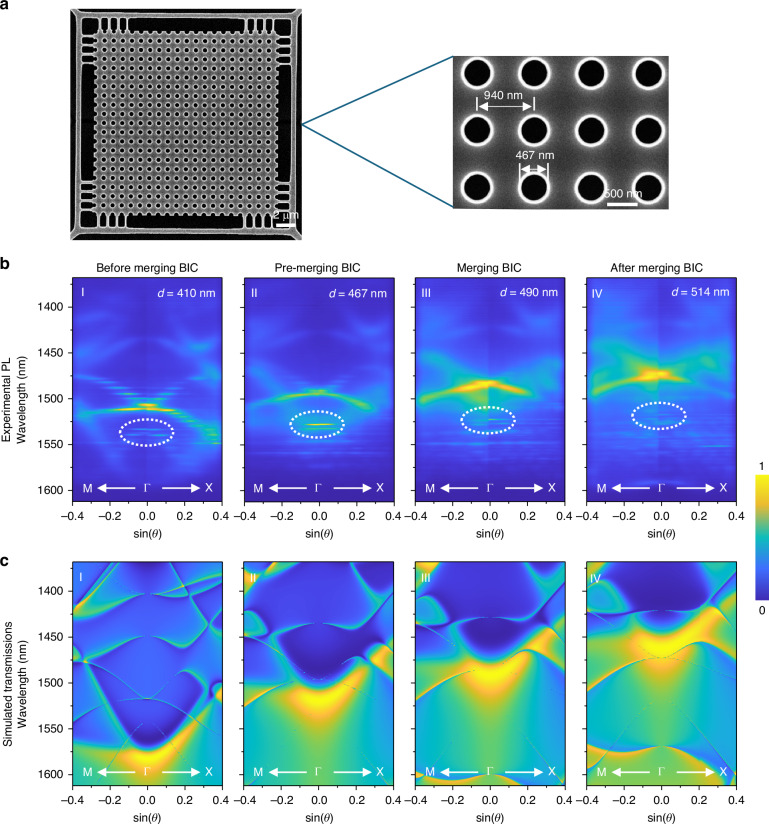
Scanning electron microscope (SEM) image and band structure analysis of the fabricated photonic crystal slabs. **a** SEM image of a photonic crystal slab with a 20 × 20 array of unit cells. The right panel shows a close-up view with a lattice constant *a* = 940 nm and hole diameter *d* = 467 nm. **b** Experimental normalized angle-resolved photoluminescence (PL) spectra for slabs with hole diameters *d* = 410, 467, 490, and 514 nm, labeled I–IV, representing conditions from before to after merging. The BIC mode is highlighted by white dashed circles. **c** Simulated normalized transmission spectra corresponding to the experimental cases in **b** calculated using rigorous coupled-wave analysis (RCWA) open-source electromagnetic solver S4^50^ with the same parameters in b, which produced the same band structure as COMSOL (Fig. S3). Due to the high quality factor of the BIC modes, these BIC modes have a near-zero linewidth. The symmetry-protected and accidental BICs can be better identified from the zoomed-in transmission spectra shown in Fig. S4

To experimentally verify this concept, we fabricated suspended photonic crystal slabs with a 20 × 20 array of unit cells. Fabrication details are provided in Supplementary Text and Fig. S1. A scanning electron microscope (SEM) image of the sample is shown in Fig. 2a, where the period is 940 nm, the quantum well layer thickness is 320 nm, and the air hole diameters range from 400 to 600 nm. (Due to the dry-etching process in the fabrication, the etched hole side wall in the quantum well is never perfectly perpendicular. So we refer to the hole diameters as effective diameter *d*, which is the average value of the hole diameters on the top and bottom surface of the quantum well). A 1064-nm pulsed fiber laser (5 ns, 50 kHz) is used to optically excite the photonic crystal cavity. Using two-dimensional k-space imaging, we measured the photoluminescence (PL) band structures of samples with varying air hole sizes, as shown in Fig. 2b. The air hole diameters range from 410 to 514 nm in Fig. 2b-I–IV, representing the progression from before merging, to pre-merging, merging, and after-merging BIC regimes, respectively. The white dashed ellipses highlight the designed photonic band, which matches the simulated band structures in Fig. 2c and Fig. 1b very well. Due to the finite size of the photonic crystal, the measured band structure exhibits discrete energy levels corresponding to different mode distributions. Similar finite-size-induced discretization of BIC bands has also been analyzed numerically in previous works^25,38,41^, where a continuous BIC band is quantized into a ladder of cavity-like s- and p-type modes in real space. Here, the two-dimensional PL band mapping in Fig. 2b directly visualizes the discretized BIC dispersion as a set of well-separated peaks in momentum space, providing experimental evidence of finite-size quantization of BIC bands.

**Fig. 3:**
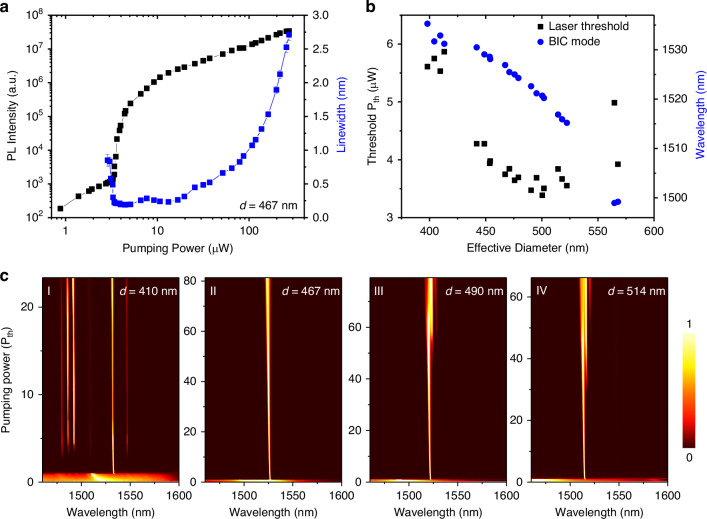
Experimental characterization of the BIC laser. **a** Log-log plot of PL intensity and the emission linewidth as a function of pumping power from a photonic crystal slab with hole diameter *d* = 467 nm, representing a slightly pre-merging condition. The linewidth of the BIC mode around the laser threshold is ~0.2 nm, corresponding to an estimated laser Q of ~7600. **b** Laser threshold and BIC mode wavelength distributions as a function of hole diameter of the photonic crystal slab, showing a reduction in laser threshold near the merging BIC condition. **c-I**–**IV** Normalized laser emission spectra as a function of pumping power for hole diameters *d* = 410, 467, 490, and 514 nm, respectively. In the pre-merging BIC condition (II), the laser achieves optimal single-mode performance, sustained up to at least 80 *P*_th_. Another set of devices also shows a similar lasing characteristics (Fig. S7)

Power-dependent micro-PL spectroscopy was performed to evaluate the impact of the merging BIC condition on the laser threshold. Figure 3a presents the power-dependent PL spectra for the sample shown in Fig. 2b-II, with an air hole diameter *d* = 467 nm, which is near the merging BIC condition. The corresponding log–log intensity plot and the observed linewidth narrowing indicate the clear laser behavior. The linewidth of the BIC mode around the laser threshold is ~0.2 nm, corresponding to an estimated laser Q of ~7600. By measuring samples with different air hole diameters, we mapped the laser thresholds and BIC mode distributions as a function of the effective hole diameter *d* (Fig. 3b), showing a distinct reduction in the laser threshold near the merging BIC condition. This observation is consistent with simulation predictions (Fig. 1) and confirms the merging BIC’s role in lowering the lasing threshold.

Upon increasing the pump power above the lasing threshold, the single-mode characteristics of different samples became evident. The BIC mode at the high-Q Γ point was consistently the first to lase, as designed. Under the before-merging condition (Fig. 3c-I), other non-BIC modes also began lasing at approximately three times the threshold power (*P*_th_), suggesting that the BIC mode did not offer clear advantages over other modes at this power. However, as the air hole diameter approached the merging BIC condition, the single-mode characteristics of the merging BIC were significantly enhanced. Surprisingly, the best single-mode performance was observed under the pre-merging BIC condition. As shown in Fig. 3c-II, lasing remained strictly single-mode even at 80× *P*_th_. In contrast, under the merging BIC condition in Fig. 3c-III, a higher-order BIC mode started to lase at approximately 50× *P*_th_. A similar trend was observed in a separate set of devices (Fig. S7).

**Fig. 4:**
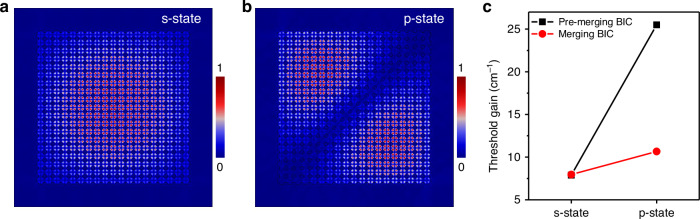
Analysis of the BIC mode in finite-size photonic crystal slabs. **a**, **b** Simulated normalized electric field distributions of the merging BIC mode in a photonic crystal slab with 20 × 20 array of unit cells. Due to restrictive boundaries, the ideal BIC band exhibits s- and p-state electric field distributions, corresponding to the discrete energy levels measured in Fig. 2b. These closely spaced energy levels result in mode competition, which directly impacts single-mode lasing performance. **c** Simulated threshold gain of the s- and p-state modes under the pre-merging and merging BIC conditions. These two modes correspond to the BIC lasing mode and the competing mode observed experimentally, i.e., the discrete peaks marked by the white dashed circles in Fig. 2b, whose energy splitting agrees well with the numerically calculated response spectra in Supplementary Fig. S8. For the pre-merging BIC, the s- and p-like modes exhibit threshold gains of 7.89 cm^−1^ and 25.5 cm^−1^, respectively, whereas for the merging BIC the corresponding values are 7.96 cm^−1^ and 10.66 cm^−1^. These results indicate that under high excitation powers, the competing mode is more likely to lase in the merging BIC scenario due to the small threshold gain difference. On the other hand, the larger threshold gain difference in the pre-merging case suggests a larger pump power range of single-mode lasing of the BIC mode, consistent with our experimental observations

This anomalous single-mode behavior is a direct consequence of the finite-sized photonic crystal slab^46^. In the 20 × 20-period devices, the BIC band exhibits discrete energy levels, as shown in the measured band diagram in Fig. 2. These energy levels correspond to distinct field distributions, with the ground state s-state and p-state depicted in Fig. 4a, b, respectively. Owing to comparable Q-factors stemming from fabrication imperfections, mode competition occurs under high excitation, which in turn influences the laser’s single-mode characteristics. To quantitatively characterize this behavior, we performed finite-difference time-domain (FDTD) simulations of the finite 20 × 20 photonic crystal array and extracted the resonance wavelength and Q-factors of the lowest-order s-like BIC mode and the first excited p-like mode under both the pre-merging and merging conditions. The underlying simulated spectra used to identify these resonances are provided in Fig. S8.

The lasing threshold gain ${g}_{{\rm{th}}}$ is defined as ${g}_{{\rm{th}}}=n/\lambda \varGamma Q$^47^, where *n* is the refractive index of the gain material, *λ* is the mode wavelength, *Γ* is the mode–gain overlap factor (~0.93 for both the BIC and competing modes), and *Q* is the mode’s quality factor. The calculated threshold gains are summarized in Fig. 4c. Under the pre-merging condition, the calculated threshold gains for the s-like BIC and the p-like competing modes are 7.89 cm^−1^ and 25.5 cm^−1^, respectively – more than a threefold difference. In contrast, under the merging condition, the threshold gains are 7.96 cm^−1^ and 10.66 cm^−1^, yielding only a ~ 1.3× difference. Although the calculated threshold-gain values under ideal conditions differ from experimentally measured threshold values due to unavoidable fabrication imperfections, the relative trends remain the same. In the pre-merging regime, the fundamental BIC mode requires substantially lower modal gain than the nearby competing modes, whereas at the merging condition the discrete s- and p-like modes exhibit much more similar threshold gain values. This occurs because merging broadens the high-Q region around the Γ point, enhancing not only the fundamental s-like mode but also nearby quantized modes such as the p-like state, thereby reducing modal discrimination in the finite lattice. In semiconductor lasers, the carrier density is strongly clamped near the threshold of the mode with the lowest ${g}_{{\rm{th}}}$; therefore, increasing the external pump power first primarily increases the photon number in that mode rather than the material gain available to competing modes. As a consequence, in the pre-merging regime, the large threshold-gain contrast prevents the p-like mode from ever reaching its higher ${g}_{{\rm{th}}}$, and strictly single-mode lasing of the s-like BIC mode is maintained even at pump powers up to 80× *P*_th_. In contrast, at the merging condition the reduced threshold-gain contrast allows the p-like mode to reach lasing at a significantly lower pump level (experimentally around 50× *P*_th_), leading to the onset of multimode emission.

These results reveal a new physical approach for robust single-mode lasing in finite-size photonic crystal BIC lasers: the pre-merging condition enhances single-mode robustness by maximizing the Q-factor contrast between the fundamental mode and nearby competitors. This effect arises from the spectral discretization and field confinement inherent in finite lattices, and is expected to occur broadly in systems exhibiting BIC merging behavior. While super-/merging-BIC concepts have largely been pursued to suppress radiative loss and reduce lasing thresholds by enhancing the radiative Q factor^39^, the primary limitation for high-power single-mode operation in finite BIC lattices is instead set by mode competition among discretized states. Crucially, we find that the optimal condition for robust single-mode operation far above threshold is not the merging regime, but rather the pre-merging regime, where the threshold gain of the nearest competing mode is maximally separated from that of the fundamental BIC mode. This counter-intuitive condition enables a substantially expanded single-mode dynamic range, reaching up to 80× *P*_th_.

**Fig. 5:**
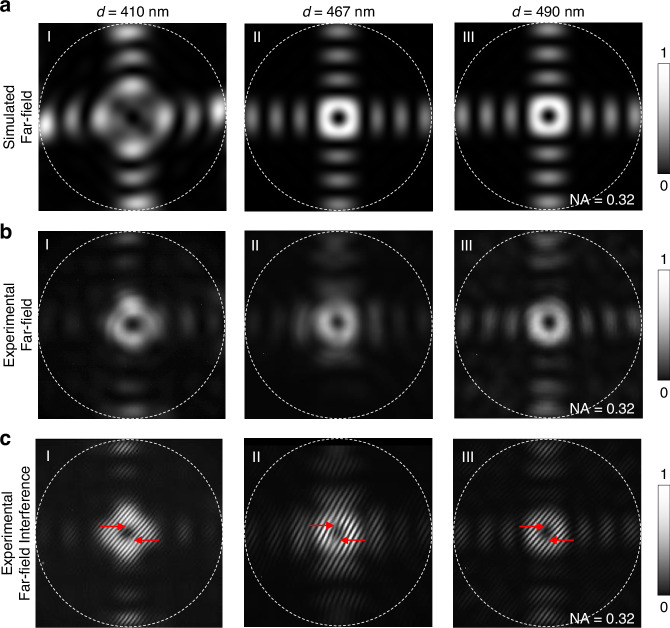
Far-field characterization of the BIC laser. **a**-**I**–**III** Simulated far-field images of the 20 × 20 array photonic crystal slabs with hole diameters *d* = 410, 467, and 490 nm, respectively. **b**-**I**–**III**, Experimental far-field images corresponding to the simulations in (**a**). **c**-**I**–**III**, Experimental self-interference patterns of the far-field images in (**b**). The double-fork patterns, highlighted by red arrows, indicate the presence of a vortex in the BIC laser emission. The white dashed circles in **a**–**c** represent the numerical aperture (NA) of 0.32

A hallmark of BIC is its topological nature, manifested as a polarization vortex in k-space^15–17^. To experimentally confirm this feature, we conducted far-field self-interference measurements using a Michelson interferometer (Fig. 5 and Fig. S5). In this configuration, the far-field emission pattern is interfered with a slightly tilted, inverted replica of itself, and phase singularities in the underlying field appear as two inverted fork-like dislocations in the interference fringes. The “double-fork” patterns highlighted by red arrows in Fig. 5c are characteristic of such phase singularities and are consistent with the unit topological charge of the polarization vortex predicted for the BIC mode in Fig. 1c and Fig. S2. Importantly, the donut-shaped far-field emission in Fig. 5 indicates that the radiation channel at Γ-point is strongly suppressed, consistent with the polarization singularity. In a practical finite-size photonic crystal slab, unavoidable symmetry breaking (finite aperture, boundary scattering, and fabrication imperfections) turns an ideal BIC into a quasi-BIC with a large but finite radiative Q^21,48^, so the out-coupled emission is dominated by small non-zero k components, naturally producing a donut-like distribution. Meanwhile, the high-contrast fringes obtained in the self-interference experiment demonstrate the high spatial coherence of the BIC laser emission, confirming its excellent lasing performance. Due to the finite size of the photonic crystal, additional interference arising from the device boundaries is visible away from the central singularity. Polarization intensity measurements are also provided in Fig. S6, which are consistent with the polarization vortex nature.

### BIC laser performance of the 5 × 5-period devices

**Fig. 6:**
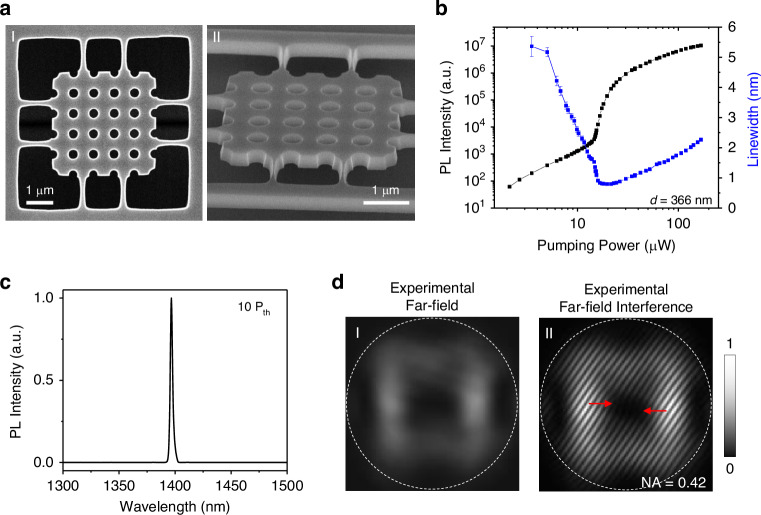
Characterization of the BIC laser from a 5×5 array photonic crystal slab. **a**-**I**–**II**, Top and side views of SEM images of the photonic crystal slab with a lattice constant *a* = 760 nm. The air-hole diameters are smaller at the edges to suppress mode leakage and enhance the Q factor. **b** Log-log plot of PL intensity and the emission linewidth as a function of pumping power from a sample with *d* = 366 nm. **c** The emission spectrum at 10× *P*_th_ of the sample in **b** which maintains single-mode performance. **d** Far-field images and self-interference patterns of the sample in (**b**). Double-fork patterns, highlighted by red arrows, indicate the vortex structure of the BIC emission. The white dashed circles denote the NA of 0.42

Despite the exceptional performance of the merging BIC laser with a 20 × 20 array, achieving BIC lasing in even smaller devices is critical for advancing miniaturized photonic technologies. Leveraging the concept of merging BIC, we also experimentally demonstrate an ultra-compact photonic crystal slab laser comprising only 5 periods, as shown in Fig. 6a. However, due to the extremely small photonic crystal size, the BIC modes experience significant leakage at the sample edges, losing their advantage over competing modes. Additionally, the highly discretized band structure in such a small device prevents continuous tuning of the merging BIC condition. To address these limitations, we employed edge-engineering by slightly reducing the air-hole diameter at the device boundaries to ~75% of that in the center, as illustrated in Fig. 6a. Simulations confirm that this edge-engineering structural optimization significantly enhances the Q-factor of the BIC mode in the 5 × 5 array compared to the unmodified BIC design, as shown in Fig. S9. Nevertheless, the increased boundary loss inherent to such small cavities inevitably leads to a higher lasing threshold. To compensate for this, we utilized the 300-nm thick quantum well gain medium and designed the BIC mode near 1400 nm, where the gain spectrum peaks under high excitation power. The resulting device has a period of 760 nm.

Power-dependent PL intensity and linewidth measurements confirm lasing behavior in Fig. 6b, with the laser maintaining single-mode operation even at excitation powers exceeding 10 times the threshold (Fig. 6c). Although the mere 5-period size prevents direct band structure measurements, far-field interference patterns reveal distinct vortex structures in momentum space, verifying that lasing originates from the BIC mode. Moreover, the small physical footprint leads to a broader distribution in momentum space, further supporting its unique BIC-based origin of the lasing mechanism. It is worth noting that previous mini-BIC lasers^38,41^ achieve footprint reduction by defining a mini-BIC cavity core within a much larger photonic-crystal heterostructure, where the surrounding photonic bandgap provides in-plane confinement. By contrast, in our approach the entire patterned photonic-crystal slab is limited to only 5 × 5 periods, and the Q enhancement is realized by direct edge engineering without increasing footprint, offering a route toward BIC single-mode lasing with the smallest possible total patterned active footprint.

## Discussion

We have demonstrated a robust single-mode laser based on the concept of merging BIC. By tuning the geometry parameters of a photonic crystal slab, multiple accidental BICs were merged with a symmetry-protected BIC, resulting in a significant reduction in the lasing threshold. More importantly, a highly robust single-mode laser operating at up to 80 times the threshold was achieved under the pre-merging BIC condition in a 20 × 20-period device, an important effect previously never discovered. In addition, we also achieved an ultra-compact photonic crystal BIC laser consisting of only a 5 × 5-period array with a patterned area of ~15 μm², much smaller than previous demonstrations^36,39,41^. This work introduces a novel approach for achieving compact single-mode lasers and offers new insights into the design of finite-size photonic crystals. Notably, the observed single-mode robustness under the pre-merging condition arises from a new but generally applicable mechanism in finite-size photonic lattices, where spectral discretization and Q-factor contrast between competing modes collectively favor the dominance of the fundamental BIC mode. For scaling to larger-area photonic crystal cavities, where mode competition becomes more stringent, edge engineering (validated in our 5 × 5 devices), introducing mode-selective defects (for loss) and optimizing the pump profile (for gain), and/or perturbed band structure^21,48^, offer practical routes to enhance mode discrimination by increasing the relative loss of higher-order modes and favoring gain for the fundamental mode. In the future, incorporating electrical pumping could enable the realization of more practical micro and nanoscale lasers^4,40,49^.

## Materials and methods

### Numerical simulation

Numerical simulations of the cavity modes were performed using the finite-element method (FEM) in COMSOL Multiphysics to calculate the band structure, Q-factor, and electric field distributions. The transmission spectrum was then computed using rigorous coupled-wave analysis (RCWA) through an open-source electromagnetic solver S4^50^, which produced a band structure that was consistent with the COMSOL results (Fig. S3). The threshold gain was simulated using the FDTD method in Lumerical. In these simulations, 80 random dipole sources were placed within the photonic crystal slab to excite the modes, and the simulated spectra responses were averaged over eight different simulations.

### Device fabrication

The clean InGaAsP/InP substrates are first deposited with 34–42 nm thick layer of SiO_2_, which serves as a hard mask for InGaAsP/InP etching, via PECVD. Next, filtered PMMA A4 950 K is spin-coated onto the substrate, and e-beam lithography (EBL) is performed at an accelerating voltage of 20 kV, aperture size of 30 µm, and working distance of 8.5 mm using a Raith 150 Two EBL system. Various EBL designs are exposed with area dose, curve dose, and line dose. After developing in cold developer (1:3 methyl isobutyl ketone (MIBK): isopropyl alcohol (IPA) at a temperature of 4­8 °C), SiO_2_ is etched by a Plasma-Therm etcher using CHF_3_: Ar plasma. Next, the PMMA is removed by a Trion Sirius-T2 RIE etcher using O_2_ plasma. Using the cleaned SiO_2_ as a mask, InGaAsP/InP is etched by a Plasma-Therm etcher using H_2_:CH_4_: Ar plasma. Next, InGaAsP is suspended by wet-etching in a diluted HCl: H_2_O solution (2.5:1). Details of the fabrication can be found in Supplementary Information Section I and Fig. S1.

### Optical measurement

All optical measurements were taken under ambient conditions. The devices were characterized by a home-built confocal micro-PL setup. A 1064-nm pulsed fiber laser with 5-ns pulse and 50 kHz repetition rate was used to pump the device through a Mitutoyo 20× or 50× objective. The laser spot size is ~20 μm and ~5 μm for the 20-period and 5-period samples, respectively. The pumping power reported in the threshold plots refers to the average power incident on the sample surface. The PL was detected by an Andor spectrometer equipped with a Princeton Instruments linear array infrared detector. The maximum spectral resolution is ~0.12 nm, estimated using a single-mode laser diode with a spectral width of 0.01 nm. A Fourier imaging configuration was used to obtain the k-space image and the band structure. The sample was mounted on a rotation stage to get the k-space dispersion along ΓX and ΓM directions. A scanning mirror was used in front of the spectrometer entrance slit to scan the momentum along the k_y_ direction. The interference fringe was measured by a Hamamatsu InGaAs camera in a Michelson interferometer configuration. Details of the measurement can be found in Figure S5.

## Supplementary information

Supplementary Information for Robust single-mode laser via merging bound state in the continuum

## Data Availability

The data supporting the findings of this study are available from the corresponding authors upon reasonable request.
