# Supplementary material for: Robust single-mode laser via merging bound state in the continuum
Source: Light Sci Appl. 2026 May 27;15:255. doi: 10.1038/s41377-026-02355-w (PMC13216272; doi:10.1038/s41377-026-02355-w)
Supplement: Supplementary file 1 — Supplementary Information for Robust single-mode laser via merging bound state in the continuum [file 41377_2026_2355_MOESM1_ESM.pdf]

Supplementary Information for

Robust single-mode laser via merging bound state in the continuum

Authors: Kai Peng[1†], Jiyoung Moon[2†], Yilin Meng[1], Kiyanoush Goudarzi[2], Wei Li[1], Qing Gu[2,3]*, Wei Bao[1]*

Affiliations:

[1]Department of Materials Science and Engineering, Rensselaer Polytechnic Institute, Troy, New York, USA

[2]Department of Electrical and Computer Engineering, North Carolina State University, Raleigh, NC 27695, USA

[3]Department of Physics, North Carolina State University, Raleigh, NC 27695, USA

[†]These authors contributed equally to this work.

*Corresponding authors. Email: baow2@rpi.edu (W.B.), qgu3@ncsu.edu (Q.G.)

The PDF file includes:

Supplementary Text

Figs. S1 to S9

Supplementary Text

Section I: Device fabrication

The schematics of the device fabrication process are shown in Fig. S1. Two types of InGaAsP/InP substrates (300 nm and 320 nm gain thickness) are first cleaned by soaking in acetone for 1 minute, followed by soaking in IPA for a few seconds, and dried with an N_2 gun. A 34-42 nm thick layer of SiO_2, in which SiO_2 serves as a hard mask for InGaAsP/InP etching, is deposited on these substrates via PECVD (Plasma-Therm 790) using 2% SiH_4:N_2O plasma (400:900 sccm), 900 mTorr pressure, and 50 W power at 250 °C. The SiO_2 thickness is verified by ellipsometry. Next, filtered PMMA A4 950K is spin-coated onto the substrates using a two-step process (500 rpm, 100 rpm s^{-1} acceleration for 5 s, followed by 3000 rpm, 3000 rpm s^{-1} acceleration for 60 s), and baked for 3 minutes at 180 °C. Next, e-beam lithography (EBL) is performed at an accelerating voltage of 20 kV, aperture size of 30 μm, and working distance of 8.5 mm using a Raith 150 Two EBL system. Various EBL designs are exposed with an area dose of 340 μC cm^{-2}, a curve dose of 240 μC cm^{-2}, and a line dose of 480 pC cm^{-1}. After developing in cold developer (1:3 methyl isobutyl ketone (MIBK): isopropyl alcohol (IPA) at a temperature of 4-8 ℃) for 30 s, the samples are rinsed by soaking in water and subsequently dried with an N_2 gun. With the developed PMMA as a mask, SiO_2 is etched by a Plasma-Therm etcher using CHF_3: Ar plasma (40:10 sccm), 800 W power, and 10 mTorr pressure at room temperature. Next, the PMMA is removed for 40 s by a Trion Sirius-T2 RIE etcher using O_2 plasma (30 sccm), 250 mTorr pressure, and 50 W power at room temperature. Using the cleaned SiO_2 as a mask, InGaAsP/InP is etched for 31 cycles (each cycle consisting of 100 s of etching time and 200 s of cooling time) by a Plasma-Therm etcher using H_2:CH_4: Ar plasma (52:9:20 sccm), 5 mTorr pressure, and 85 W power at 50 °C. Next, InGaAsP is suspended by wet-etching in a diluted HCl: H_2O solution (2.5:1). The samples are immersed in the diluted HCl solution for tens of seconds at a time. The suspension process is monitored under an optical microscope and top-view SEM between the wet-etching intervals. Note that a higher concentration of HCl and/or a prolonged wet-etching time can distort the circular shape into an angular shape. After rinsing with water, the SiO_2 mask is removed by soaking in BOE 7:1 for 3 minutes.

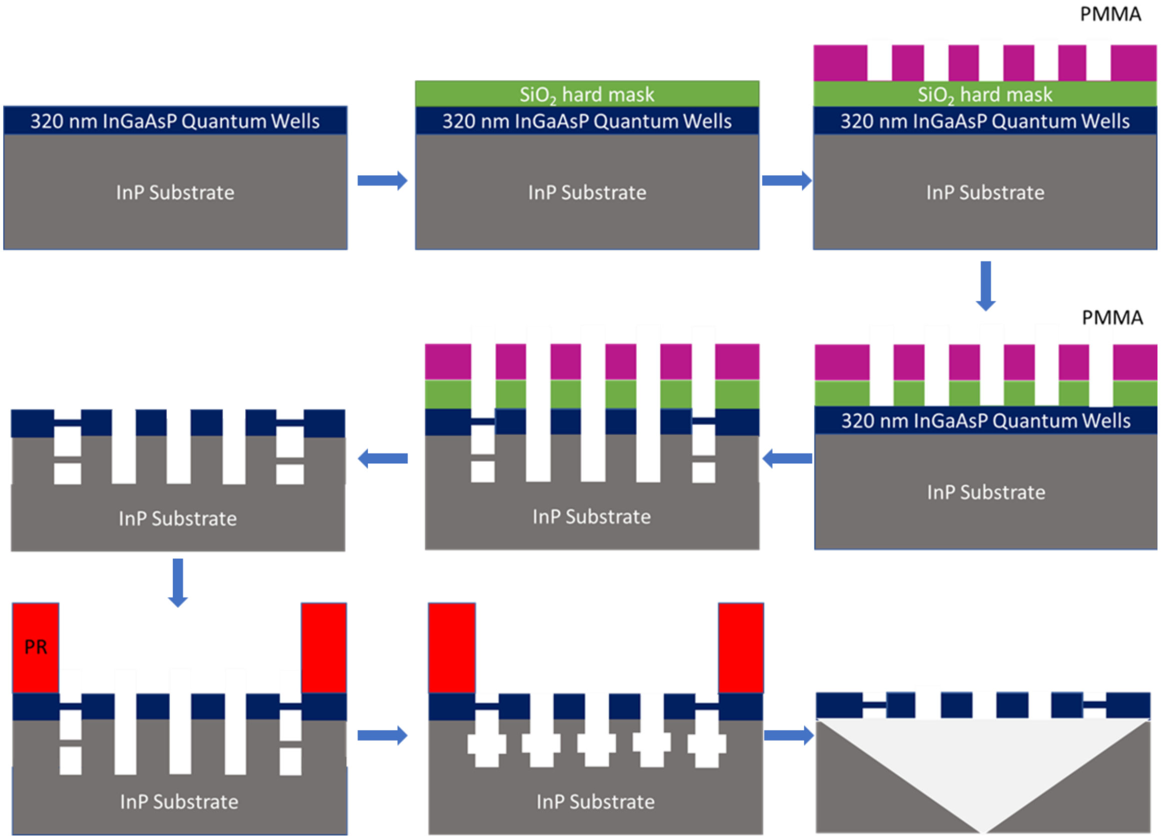

Fig. S1 Schematic illustrations of the fabrication process. The BIC laser devices are fabricated by standard nanofabrication techniques. Details can be found in Supplementary Text Section I.

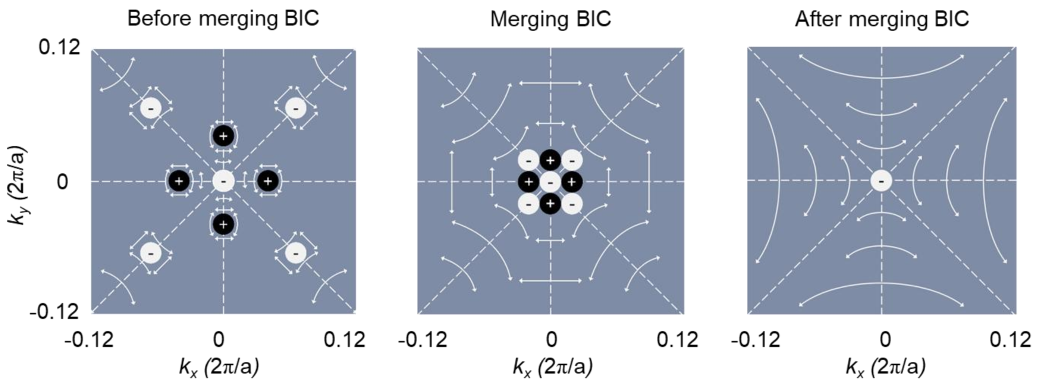

Fig. S2 Illustration of the topological charge distributions of the BIC modes in momentum space. Three geometric conditions considered are before merging, near (or pre-) merging, and after merging. The black and white circles denote topological charges of opposite signs (±1), and the arrows indicate polarization vortices. As the structural parameters (e.g., air-hole diameters) vary, off-Γ accidental BICs gradually move toward the central symmetry-protected BIC at Γ and eventually merge to form a single, merged BIC. This process manifests as the coalescence of topological charges, consistent with the Q-factor evolution shown in Fig. 1c.

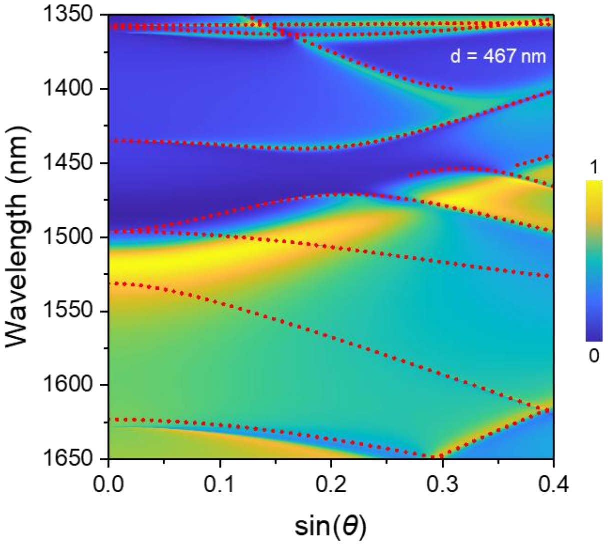

Fig. S3 Comparison of the photonics band structures calculated via two methods (S4 and COMSOL) for the same photonic crystal slab ($d = 467$ nm, corresponding to Fig. 2b-II). The colormap shows the transmission spectrum along ΓX calculated by S4, and the red solid dots represent the high-Q ($Q > 100$) eigenvalues obtained from COMSOL. Close agreement can be seen.

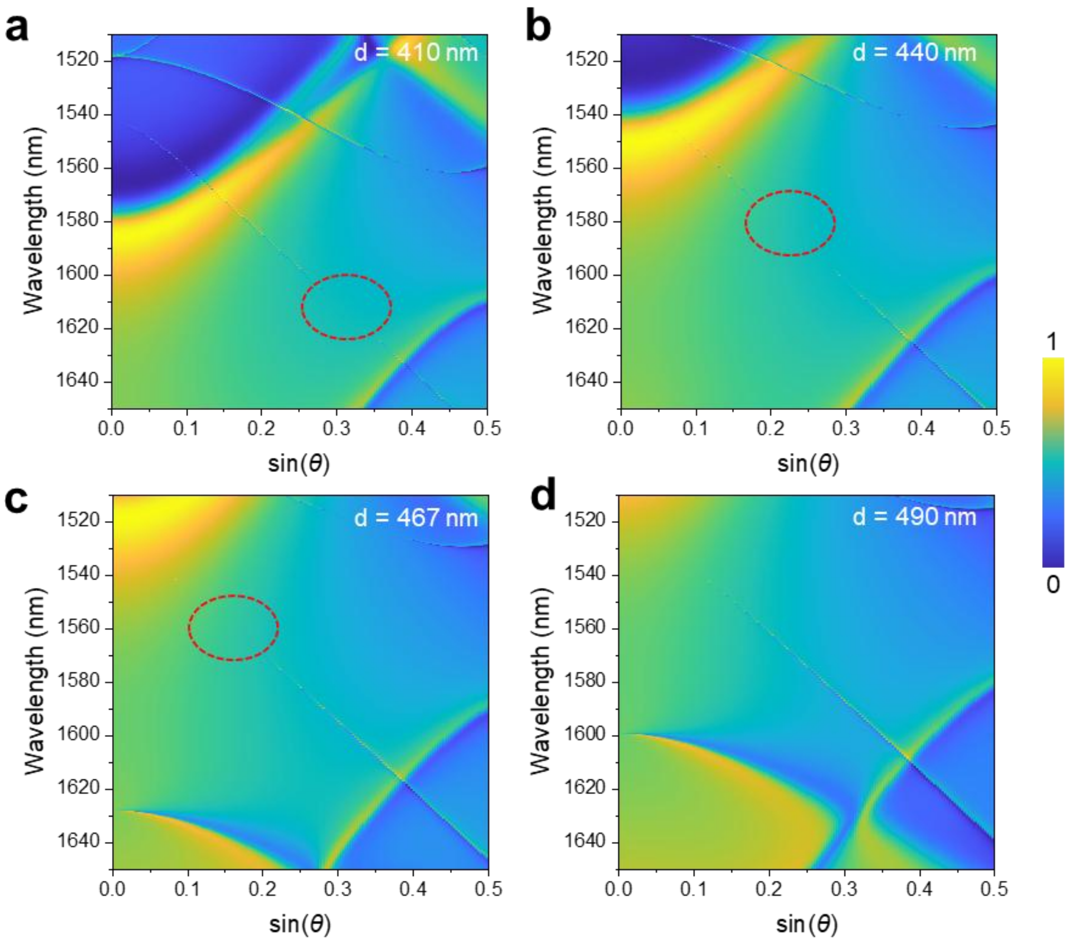

Fig. S4 Simulated normalized transmission spectra along ΓX by S4. Light incident on the photonic crystal slab excites guided resonances, resulting in sharp Fano features in the transmission/reflection spectra. In contrast, a perfect BIC mode exhibits no Fano features due to its complete decoupling from the radiation field, which manifests as the disappearance of the transmission/reflection spectra. As highlighted by the red ellipses in the figure, in the before-merging BIC condition, namely, **a,** d = 410 nm, **b,** d = 440 nm, and **c,** d = 467 nm, accidental BICs appear at non-Γ points, leading to spectral disappearance, which is consistent with the Q-factor distribution calculated using COMSOL in Fig. 1. As the air-hole diameter increases, the accidental BIC gradually shifts towards the Γ point and eventually merges in the merging BIC condition at **d,** d = 490 nm.

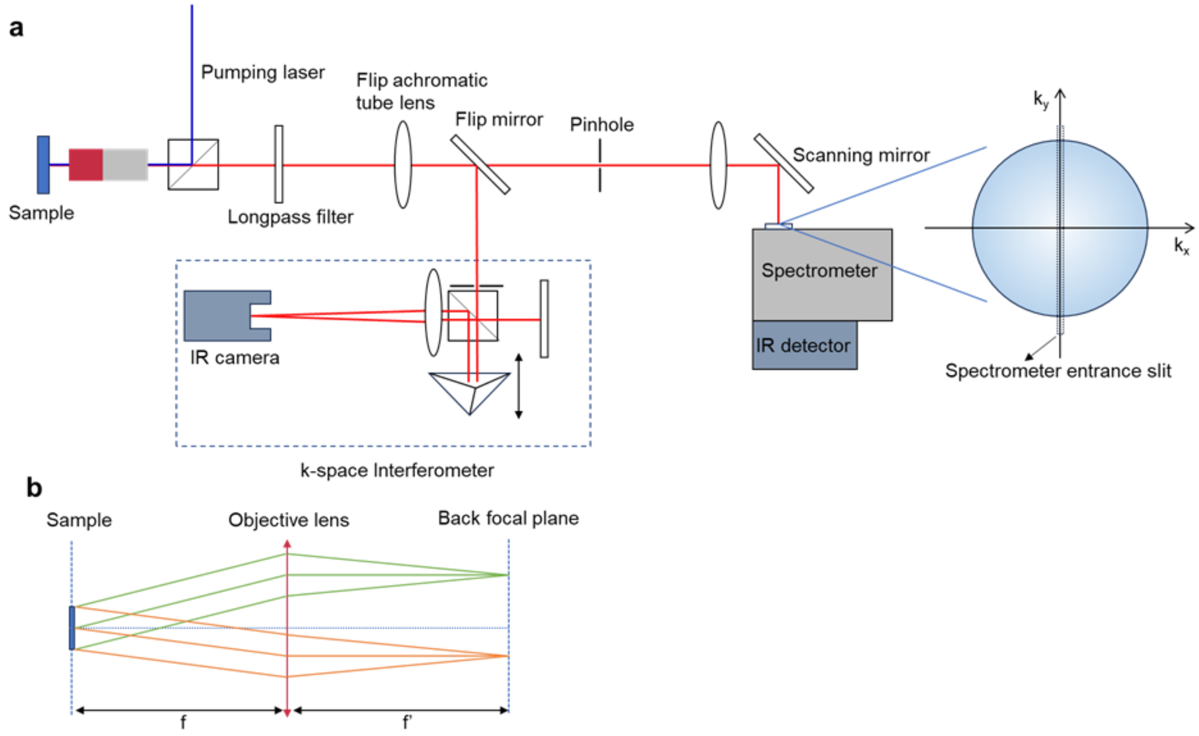

Fig. S5 Measurement setup schematic. a, Experimental setup for infrared k-space band-mapping and self-interference measurement. The sample was optically pumped by a 1064-nm pulsed fiber laser (pulse width: 5 ns, repetition rate: 50 kHz). The PL was collected in a reflection configuration through a Mitutoyo 20× (numerical aperture: 0.4) or a 50× (numerical aperture: 0.42) objective and analyzed using an Andor spectrometer equipped with a Princeton PyLoN infrared detector. The maximum spectral resolution is ~0.12 nm. For k-space imaging, a Fourier imaging configuration with two tube lenses was utilized. The linear array of the IR detector in the spectrometer cannot directly obtain the two-dimensional k-space image. Therefore, to measure the band structure, a scanning mirror was positioned in front of the spectrometer entrance slit. By scanning the mirror along the vertical direction, the spectra along the k_y direction can be recorded by the spectrometer and the linear IR detector. Additionally, by rotating the sample, measurements along various momentum directions, as shown in Fig. 2 and Fig. S7, were obtained. Interference patterns were measured using a Hamamatsu InGaAs camera integrated into a Michelson interferometer. The interferometer was carefully aligned to achieve the k-space image interference, as demonstrated in Fig. 5. **b**, The schematic of the back focal plane (BFP) of the objective lens. Here, f and f' represent the focal lengths before and after the objective lens. The rays emitted at the same angle are focused to the same lateral position on the BFP, as illustrated by the green and orange rays. Consequently, each point on the BFP corresponds to the superposition of the optical field emitted into a specific direction.

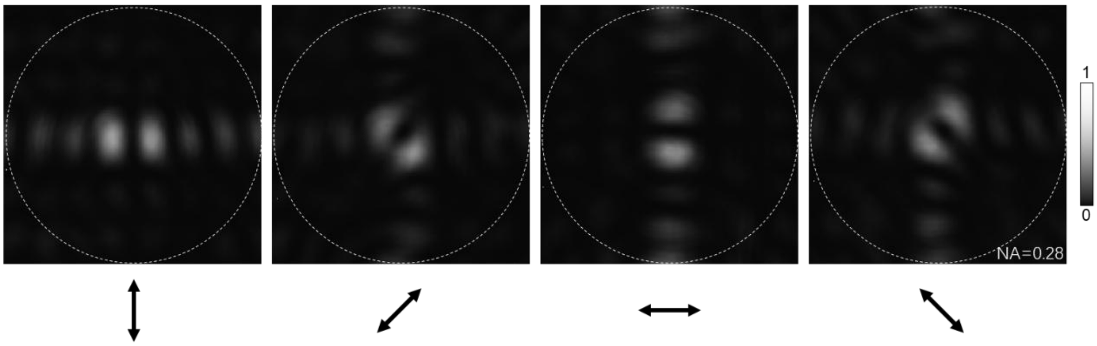

Fig. S6 Experimental polarization-resolved k-space images of the BIC laser emission. A polarizer was inserted before the IR camera for the sample with d = 490 nm in Fig. 5b-III. The arrows below the images represent the linear polarization direction. This polarization-resolved lasing intensity distribution illustrates the vortex nature of the BIC laser, consistent with the interference experiment in Fig. 5c-III.

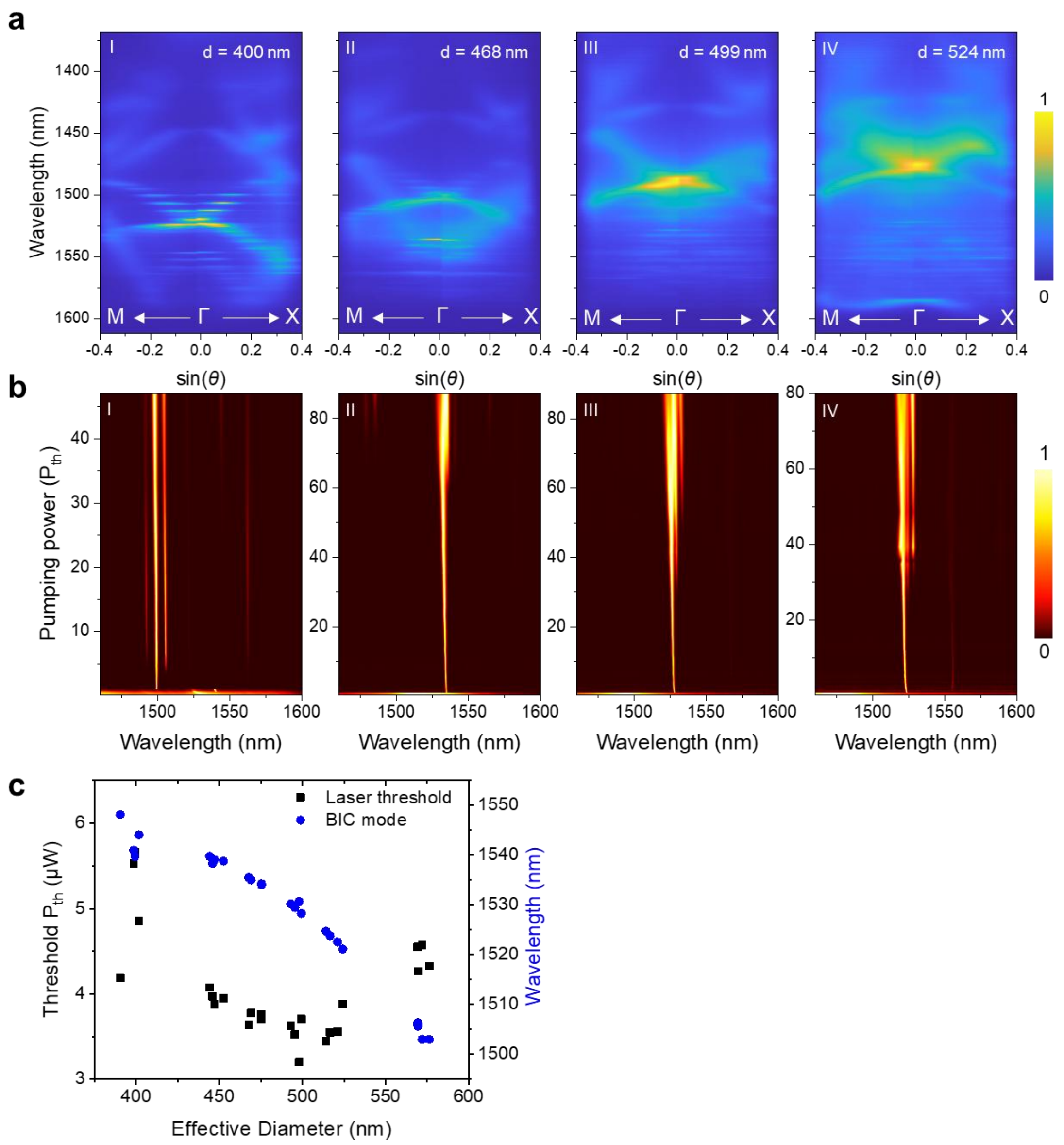

Fig. S7 Laser performance of another set of photonic crystal slabs with 20×20 arrays. Because of fabrication variations between different batches of samples, this set of samples shows band structure a little different from the devices in Fig. 2-3. **a-I-IV**, Experimental angle-resolved PL spectra for slabs with hole diameters d = 400, 468, 499, and 524 nm, labeled I-IV, representing conditions from before to after merging. **b-I-IV**, Normalized laser emission spectra as a function of pumping power for the samples in **a-I-IV**, respectively. In the pre-merging BIC condition (II), the laser achieves optimal single-mode performance, sustained up to at least 60× P_{th}, showing better single-mode performance than other conditions and consistent with the result in the main text. **c,** Laser threshold and BIC mode distributions as a function of effective hole diameter in the photonic crystal slab, showing a reduction in laser threshold near the merging BIC condition.

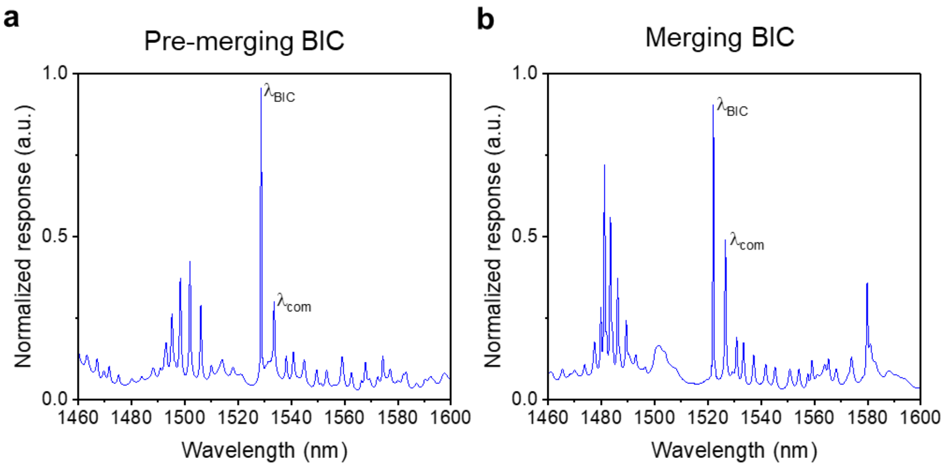

Fig. S8 Simulated normalized mode responses under the pre-merging and merging BIC conditions. The mode separations between the BIC (λ_{BIC}) and competing mode (λ_{com}) are 4.85 nm and 4.52 nm in **a** and **b**, closely matching the experimental values of 4.63 nm and 4.4 nm observed in Fig. 2b-II and 2b-III. Details of the FDTD calculation can be found in the Materials and methods section.

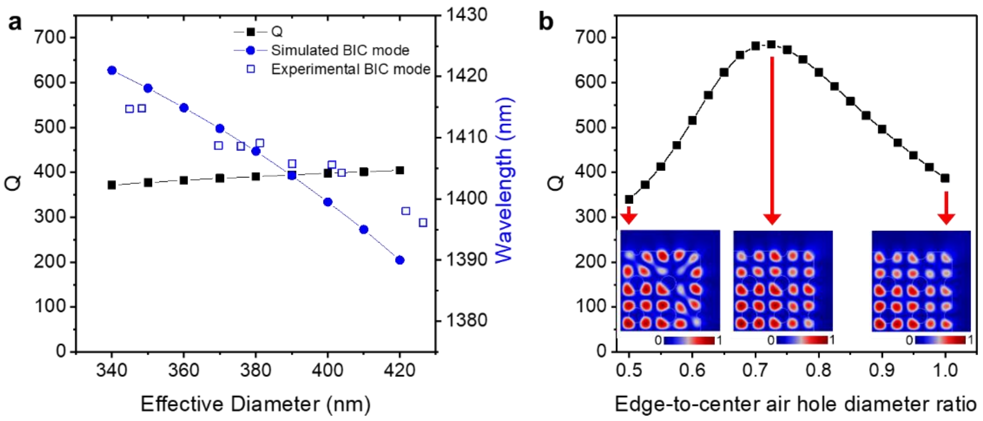

Fig. S9 Simulation of edge modification of the photonic crystal slab of 5×5 arrays. Due to the limited number of periods, the photonic band structure becomes highly discretized in k-space, making it impossible to resolve a continuous band. As a result, the mechanism for merging BIC cannot be established. In this regime, radiation leakage is dominated by edge effects rather than internal interference-based BIC protection, leading to a Q factor that is largely insensitive to structural parameters such as the air hole diameter. **a**, Although the BIC mode wavelength varies continuously, the Q factor remains nearly constant. However, edge engineering can effectively suppress radiation loss and enhance the Q factor. In our design, the diameters of edge holes were systematically reduced while keeping all other parameters unchanged. **b**, For a structure with a central hole diameter of 370 nm, the simulated Q factor reaches a maximum when the edge hole diameter is about 73% of the center holes. The corresponding electric field distribution becomes more uniform, as shown in the inset. The simulations were carried out using a full 3D model in COMSOL Multiphysics, where each air hole was modeled as a truncated cone with sidewalls tilted by ~9° with respect to the vertical axis, reflecting the actual etching profile. The effective diameter was used to represent the lateral size of the air hole at the central quantum well layer. The surrounding medium was set to air, and scattering boundary conditions were applied to simulate radiation into the open space. The complex eigenfrequencies of the BIC modes were calculated using the eigenfrequency solver, from which both Q factors and electric field profiles were extracted. The fabricated sample has edge hole diameters of approximately 75% of the central holes, in agreement with our design.
